# Using Unified Theory of Acceptance and Use of Technology to Evaluate the Impact of a Mobile Payment App on the Shopping Intention and Usage Behavior of Middle-Aged Customers

**DOI:** 10.3389/fpsyg.2022.830842

**Published:** 2022-03-03

**Authors:** Che-Hung Liu, Yen-Tzu Chen, Santhaya Kittikowit, Tanaporn Hongsuchon, Yi-Jing Chen

**Affiliations:** ^1^Department of Business & Management, National University of Tainan, Tainan, Taiwan; ^2^Department of Information and Learning Technology, National University of Tainan, Tainan, Taiwan; ^3^Chulalongkorn Business School, Chulalongkorn University, Bangkok, Thailand

**Keywords:** unified theory of acceptance and use of technology (UTAUT), degree of involvement, mobile payment, middle-aged customers, shopping intention

## Abstract

This research adopted the unified theory of acceptance and use of technology (UTAUT) to emphasize the use of the PX Pay mobile payment app for PX Mart, the most popular supermarket in Taiwan, and examine the degree of involvement as a moderator. The influence of factors related to PX Mart’s target customer groups on their shopping intentions and usage behaviors were discussed, with subsequent benefits and optimization directions. This study indicated the following results. First, performance expectations, ease-of-use expectations, and social impact enhance consumers’ behavioral intention and behavioral intention significantly influence usage behavior. Second, gender has no significant moderating effect on the proposed model. Third, age has a moderating effect from performance expectations and ease-of-use expectations, social influence on behavioral intention. Fourth, use is more significantly affected by perceived stakeholders for customers over age 50 than for those age 30–39. Fifth, the degree of involvement significantly affects the relationship between behavioral intention and usage behavior in terms of social influence and facilitating conditions effects. Finally, we provided academic and practical implications and make contributions to both the online payment industry and academia.

## Introduction

Mobile payment has been witnessed in all areas of daily consumption for people in Taiwan. According to statistics from the Taiwan Industry Department of the National Development Commission, the penetration rate of mobile payments in 2019 was 62.2% and may achieve the ultimate goal of 90% in 2025 (National Development Commission, 2020). Currently, payment service providers are not limited to the traditional financial industry. Major convenience stores in Taiwan have launched their own mobile payment systems, such as My FamiPay, OPEN Wallet, HiPay, and OK Pay. The flourishing development of mobile payment systems has received strong support from software service providers, mobile phone manufacturers, social platforms for the traditional retail industry, and the mobile payment market (Iman, 2018). According to the statistics by the Council for Information, mobile payments increased by 42.2% during the epidemic, with the largest increase among mainstream payment tools, followed by physical cards (increased by 35.2%) and electronic tickets (increased by 28.7%); in contrast, the frequency of cash users decreased by 33.4%, showing that the epidemic has gradually changed consumers’ payment patterns and accelerated the development of consumers’ non-contact consumption habits (Commercial Times, 2021).

Many studies have examined the impact of mobile payments on consumer behavior (Schierz et al., 2010; Arvidsson, 2014; Oliveira et al., 2016; de Luna et al., 2019; Lin et al., 2019). Mallat and Tuunainen (2005) proposed that mobile payments enable consumers to complete the payment process more conveniently, to reduce payment costs, and to increase consumer impulse purchases. Therefore, mobile payments may increase the probability of increasing the sales rate. When discussing the user adoption of new technologies, most studies are based on the technology acceptance model (TAM) or the subsequent unified theory of acceptance and use of technology (UTAUT) to explore the use of mobile payments (Slade et al., 2014, 2015; de Sena Abrahão et al., 2016; Patil et al., 2020). More than 60% of PX Mart customers in the case of this study are traditionally recognized as a group with relatively poor use of electronic payment tools due to the middle to higher age range of the target customers.

Most studies explore the acceptance of electronic payment tools only by young people and seldom study the consumption behavior of middle-age to older people (Fernández-Ardèvol and Prieto, 2012; Iancu and Iancu, 2020). PX Mart, the largest Taiwanese supermarket with more than 1,000 stores, announced its PX Pay mobile payment system last year and became one of the most powerful mobile payment systems in Taiwan. This research focused on the target customer groups’ experience in using PX Pay, explored their behavioral intentions, and then sought to understand the relationship between shopping behavior and acceptance. The specific research goals are to (1) evaluate the impact of different dimensions on consumers’ intention to use PX Pay and understand the factors and main purposes for using PX Pay for middle-aged consumers; (2) explore how the demographic variables including gender and age affect the acceptance of PX Pay by middle-aged consumers; and (3) understand PX Pay use experience and involvement to impact middle-aged consumers’ acceptance of PX Pay.

## Literature Review and Theoretical Background

### Unified Theory of Acceptance and Use of Technology

Studies based on UTAUT have found that its ability to explain technology usage behavior is much stronger than the ability of any single model as an extended model of other technology acceptance models (AbuShanab and Pearson, 2007; Im et al., 2011; Venkatesh et al., 2011, 2012; Williams et al., 2015; Lin et al., 2019; Alghazi et al., 2021; Andrews et al., 2021; Arfi et al., 2021; Bu et al., 2021). Variables including use performance expectations, ease-of-use expectations, social influences, and facilitating conditions are used to explore behavioral intentions and usage behaviors. Moreover, four possible moderating variables are proposed, namely, gender, age, experience and voluntary use, to explore usage behavior. Since UTAUT has a high degree of explanatory power for people’s intentions and behaviors in using technology, this research used the UTAUT framework to analyze the influence of middle-aged PX Mart customers’ behavioral intentions to use PX Pay and related factors. Performance expectations, ease-of-use expectations, social impact and facilitating conditions are the independent variables in this study. Consumers may have different behavioral intentions and usage behaviors when using PX Pay.

### Involvement

Involvement is a response to an inner state (Mitchell and Olson, 1981). Consumers pay attention to product information due to a specific situation or stimulus. Zaichkowsky (1985) broadly interpreted involvement as referring to a person’s subjective perception of the importance of something based on his or her own values, interests, and needs and claimed that according to the different objects involved, involvement is divided into advertising involvement, product involvement, and purchase involvement. Laurent and Kapferer (1985) emphasized that consumers with high involvement tend to adopt a rational decision-making model, while consumers with low involvement prefer simplified decision-making. Prior research also pointed out that consumers may not necessarily undertake sufficient and rational information collection, screening, and evaluation for certain products, advertisements or purchase behaviors. Involvement here plays an important role in this decision-making process. Sharma (2008) emphasized that the degree of involvement reflects the personality traits surrounding consumption and affects information collection before purchase and evaluation activities after purchase, so the degree of involvement also affects consumers’ purchase intentions. In the past, the degree of involvement was mostly used to explore the purchase behavior of consumers. According to the research of Cai et al. (2017), the degree of involvement also had a significant positive impact on attitudes toward the use of new technologies. When people are willing to spend more time understanding and participating in related activities, this higher degree of involvement leads to a greater willingness to use products/services. This research concerns product involvement.

## Research Methods

### Research Structure and Hypothesis

**FIGURE 1:**
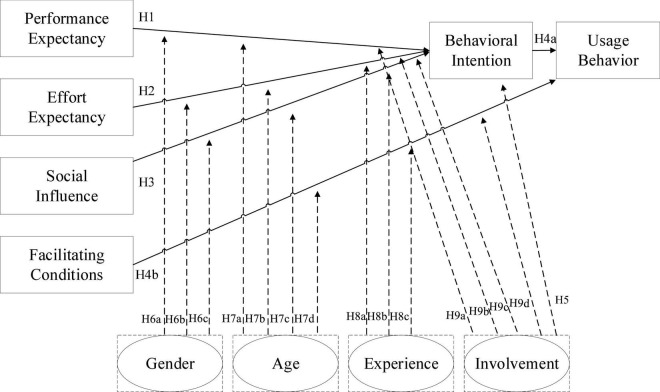
Research framework.

The variable voluntary use in the UTAUT model was removed since this study considered only consumers’ voluntary use of PX Pay of PX Mart. When prior studies have used UTAUT as a research framework for technology acceptance, the degree of involvement is rarely added in an extended discussion of factors affecting usage to explain consumers’ motivations for using PX Pay and the degree of influence on shopping intentions. Therefore, the research framework is shown as Figure 1.

Performance expectancy (PE) refers to the user’s personal perception, which contributes to user work performance (Venkatesh et al., 2003). Prior studies have pointed out that performance expectations are an important factor in the acceptance of technology (Davis, 1989; Venkatesh and Davis, 2000; Oechslein et al., 2014). When consumers can use technology to accomplish their goals, their practical and convenient expectations are met, and they are more willing to use information systems. In addition, performance expectations vary depending on gender and age (Cabrera et al., 2009; Lian and Yen, 2014). For example, men and young people pay more attention to performance expectations for information systems. Therefore, Hypothesis 1 is proposed as follows.

H1: Performance expectations positively influence consumers’ intention to use PX Pay.

Effort expectancy (EE) refers to the degree to which the user can easily operate new technologies, systems, and applications (Venkatesh et al., 2003). Ease-of-use expectations vary with gender and age (Venkatesh, 2000). For example, women and elderly people usually pay more attention to the ease-of-use expectations of information systems, but its influence is still affected by the accumulation of experience. At the same time, Maduku et al. (2016) pointed out that if consumers think that mobile marketing tools are simple and easy to use, they do not require additional time and effort, and they can find the information they need from the tools, which may increase their acceptance. Therefore, we proposed the following research hypothesis.

Hypothesis 2 (H2): Ease-of-use positively influences consumers’ intention to use PX Pay.

Social influence (SI) refers to the user’s personal knowledge that important others believe that the user should use this new technology or system (Venkatesh et al., 2003). Social impact also varies depending on factors such as gender, age, experience, and willingness (Ye et al., 2020). For example, young women and early adopters are susceptible to the influence of their senior supervisors and colleagues. Therefore, this study proposed the third hypothesis as follows.

Hypothesis 3 (H3): Social influence positively influences consumers’ intention to use PX Pay.

Facilitating conditions (FC) are helping conditions that promote the use of information technology by users (Venkatesh et al., 2003). The software and hardware resources that support users in operating a system must first be provided to solve their problem. It is expected that users will actually use information technology (Moore and Benbasat, 1991; Thompson et al., 1991). Therefore, facilitating conditions are defined according to the user’s perception along with the organization’s support for new technologies and systems. Past empirical evidence has found that facilitating conditions affect consumers’ technology adoption behavior (Venkatesh et al., 2012) and that age affects cognition (Morris and Venkatesh, 2000). At the same time, organizational psychologists have indicated that older people focus more on obtaining support and help at work, so age is one of the variables related to facilitating conditions. In addition, as use experience increases, the impact on facilitating conditions will also increase (Bergeron et al., 1990). Therefore, Hypothesis 4 is developed as follows.

Hypothesis 4 (H4): Facilitating conditions positively affect the consumption behavior of PX Pay users.

Involvement refers to consumers linking products with their subjective consciousness, showing their needs, interests, and values for the product; thus, how to improve consumer product involvement is the goal of marketing strategy design (Warrington and Shim, 2000). Knox and Walker (2003) found that consumers’ involvement is reflected in the collection of information on brand products, and the greater their involvement, the higher their loyalty toward a brand. This research defines the degree of involvement as the degree to which an individual’s perception of a product is related to its intrinsic needs, interests, and values, with the collection of data as a consideration. Therefore, Hypothesis 5 was developed as follows.

Hypothesis 5 (H5): The degree of involvement positively affects consumers’ behavioral intentions toward the use of PX Pay.

The conceptual definition of behavioral intention refers to the subjective probability of an individual’s desire to engage in a certain behavior (Ajzen and Fishbein, 1975), and many scholars (Mathieson, 1991; Szajna, 1996; Hu et al., 1999) have noted that behavioral intention can be used to empirically predict system use. Venkatesh et al. (2003) found that behavioral intentions affect consumers’ technology adoption behavior. Thus, Hypothesis 4a was developed: Behavioral intentions positively affect consumers’ behavior in using PX Pay. Given the discussion above, several hypotheses about moderating effects have been developed as follows.

H6a: Gender has a moderating effect on performance expectations and behavioral intention related to PX Pay.
H6b: Gender has a moderating effect on ease-of-use expectations and behavioral intention related to PX Pay.
H6c: Gender has a moderating effect on social influence and behavioral intention related to PX Pay.
H7a: Age has a moderating effect on performance expectations and behavioral intention related to PX Pay.
H7b: Age has a moderating effect on expectations of ease of use and behavioral intention related to PX Pay.
H7c: Age has a moderating effect on social influence and behavioral intention related to PX Pay.
H7d: Age has a moderating effect on PX Pay’s facilitating conditions and usage behavior related to PX Pay.

Hypothesis of the moderation effect of use experience: Venkatesh et al. (2003) studied use experience and found that the influence of ease-of-use expectations on behavioral intention changes with the accumulation of use experience; in particular, use experience increases along with these two variables. The effect of social influence on behavioral intention is no longer significant, and the influence of facilitating conditions on usage behavior increases with as use experience increases. Kim et al. (2005) also concluded that use experience is defined as consumers obtaining or accumulating knowledge from relevant experience and that measuring consumers’ degree of use based on frequency and time can aid in understanding behavioral willingness. Hence, Hypothesis 8 was developed as follows:

H8a: Use experience has a moderating effect on expected ease of use and behavioral intention related to PX Pay.
H8b: Use experience has a moderating effect on social influence and behavioral intention related to PX Pay.
H8c: Use experience has a moderating effect on facilitating conditions and usage behavior related to PX Pay.

In addition, according to Xiao (2008), in a study of blog users paying to purchase value-added services, users with high product involvement are very concerned about whether the service function can improve performance and are vulnerable to influence from relatives, friends or social environments. In addition, Park and Keil (2019) emphasized that the degree of product involvement moderates the relationship between helping conditions and usage behavior. Thus, the following hypotheses have been developed:

H9a: The degree of involvement has a moderating effect on performance expectations and behavioral intention related to PX Pay.
H9b: The degree of involvement has a moderating effect on ease-of-use expectations and behavioral intention related to PX Pay.
H9c: The degree of involvement has a moderating effect on social impact and behavioral intention related to PX Pay.
H9d: The degree of involvement has a moderating effect on facilitating conditions and usage behavior related to PX Pay.

### Selection of the Research Samples and Operational Definition and Design of Questions

Before PX Mart developed PX Pay, the main customer ages were from 40 to 59 years old (55%), and more than half were women. After PX Pay was launched, the proportion of customers over age 40 increased by 60%, customers over 50 accounted for 26%, and female customers accounted for more than 70% of overall use. Meanwhile, PX Mart observed that the most popular age group for PX Pay was between 30 and 49 years old. In view of the close interaction and connectivity between age groups and PX Mart, the selection of research subjects mainly focuses on (1) individuals over 30 years old and (2) users who have downloaded and used PX Pay.

This questionnaire was divided into four parts with a total of 39 questions summarizing the use of PX Pay, use factors and intentions, subjective awareness and actual perception of personal involvement, and basic personal information. A small pre-sample survey was conducted, and a total of 53 responses were collected. After reliability and validity analysis, 7 items were deleted, and then the questionnaire was reorganized.

## Empirical Analysis and Results

### Sample Structure and Narrative Analysis

A total of 326 questionnaires were collected, and the final accepted number was 259 because some questionnaires did not meet the eligibility requirements. The statistical results of the narrative analysis were as follows. The proportion of women (188) was 72.6%, and the share of men (71) was 27.4%. Age 30–39 accounted for 35.9% of the sample, age 40–49 accounted for 38.2%, and age 50 and older accounted for 25.9%. In addition, 77.2% of participants were married, and 22.8% were single. In terms of education level, most (85.7%) had received a bachelor’s degree. Overall, their characteristics corresponded to the profiles of PX Mart’s statistics on PX Pay users.

According to the statistical results of analyzing PX Pay use experience, the frequency of PX Pay users shopping on PX Mart shows a normal distribution, mostly concentrated in “sometimes (2–3 times a week)” and “occasionally (once per week).” In terms of using different carriers to collect points, more than 84.9% of consumers would use PX Pay to collect points when shopping at PX Mart, and a small number would interact with physical welfare cards. Regarding frequency of use, 79.5% said they “use every time,” and 10.8% said “will use 80% of the time”; thus, the subjects used it relatively frequently. When asked about the PX Pay functions and operations, 40.2% found it “understandable” and 29.3% found it “very understandable.” In total, nearly 70% of users had a certain degree of mastery and familiarity with the use of PX Pay.

According to the findings of this research, the important factors that make consumers willing to use PX Pay were to “save time and speed up checkout,” “convenient management of invoices and consumption records” and “returns and discounts.” The more frequently used and recommended functions by PX Pay users were “accumulated points,” “binding credit card payment,” “consumption history view,” “winning information” and “batch shopping/pickup.”

A reliability and validity analysis were then conducted. The CR of the overall research dimension was between.802 and.959, and the AVE was between.526 and.824, which is acceptable to measure the reliability of the variable (as shown in Table 1). Moreover, discriminant validity was assessed, and both a facet correlation matrix and cross-loading matrix confirmed that the various aspects of this study had acceptable discriminant validity (as shown in Table 2).

**TABLE 1:** Reliability and convergent validity.

Construct	Scale	Mean	Standard deviation	Composite reliability (CR)	Average variance extracted (AVE)
Performance expectancy Cronbach’s α = 0.93	PE1	4.14	0.88	0.94	0.78
	PE2	4.21	0.75		
	PE3	4.21	0.71		
	PE4	4.07	0.80		
	PE5	4.08	0.76		
Effort expectancy Cronbach’s α = 0.97	EE1	4.01	0.77	0.96	0.82
	EE2	4.21	0.72		
	EE3	3.92	0.81		
	EE4	4.00	0.76		
	EE5	4.07	0.75		
Social influence Cronbach’s α = 0.83	SI1	3.35	0.84	0.87	0.53
	SI2	3.19	0.96		
	SI3	3.90	0.82		
	SI4	2.91	1.05		
	SI5	2.86	1.06		
	SI6	3.05	1.07		
Facilitating conditions Cronbach’s α = 0.83	FC1	3.89	0.77	0.89	0.74
	FC2	3.89	0.80		
	FC3	3.97	0.74		
Behavioral intention Cronbach’s α = 0.91	BI1	4.31	0.64	0.94	0.78
	BI2	3.95	0.77		
	BI3	4.03	0.75		
	BI4	4.23	0.65		
Experience Cronbach’s α = 0.52	Exp1	4.70	0.63	0.80	0.67
	Exp2	3.98	0.80		
Involvement Cronbach’s α = 0.87	Inov1	3.63	0.87	0.92	0.79
	Inov2	3.34	0.98		
	Inov3	3.31	0.99		
Usage behavior Cronbach’s α = 1.00	UB1	3.32	0.61	1.00	1.00

**TABLE 2:** Discriminant validity.

Construct	PE	EE	SI	FC	BI	UB
Performance expectancy	***0.88***					
Effort expectancy	0.80	***0.91***				
Social influence	0.44	0.47	***0.73***			
Facilitating conditions	0.59	0.69	0.41	***0.86***		
Behavioral intention	0.68	0.64	0.42	0.69	***0.88***	
Usage behavior	0.24	0.23	0.21	0.23	0.25	***1.00***

PE, performance expectancy; EE, effort expectancy; SI, social influence; FC, facilitating conditions; BI, behavioral intention; UB, usage behavior. The values on the diagonal are the square root of the AVE value of each construct, and the values on the non-diagonal are the correlation coefficient.

### Structural Model Analysis and Hypothesis Test Results

SmartPLS was adopted in this research to implement the least square method in the structural equation, with the correlation test of the path coefficient (ß value) of each aspect of the research model, and the *R*^2^ is used to determine the predictive power of the model and the variables. This approach is the basis of causal mode analysis over time to identify whether the corresponding path has a significant relationship, strength and direction and then to determine whether the hypothesis is supported by the data.

Then, SPSS was adopted to perform hierarchical regression analysis to verify the hypothesis of the effects of variables in the research framework and to explore the effect of each moderating variable, and then independent-sample t-test and single-factor variance analysis are used to help explain the key elements of these effects. The description of the statistical analysis is divided into two main axes.

#### Model Analysis Results

**FIGURE 2:**
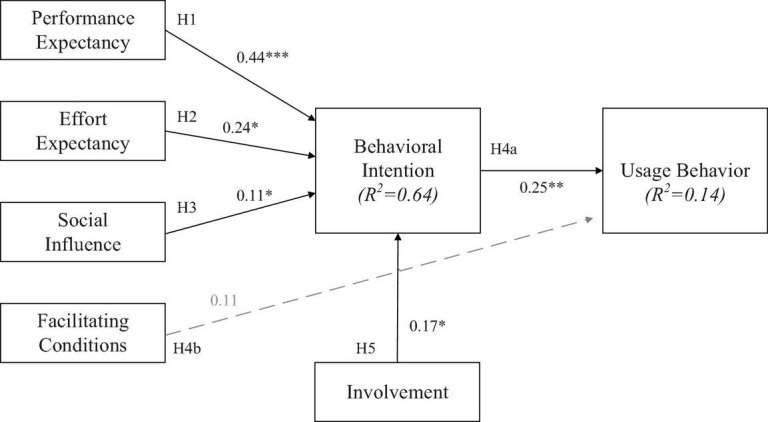
Path coefficients of the structural model. *p < 0.05; **p < 0.01; ***p < 0.001.

Performance expectations had a significant positive effect on consumers’ behavioral intention to use PX Pay (β = 0.44, *t*-value = 6.16, *p*-value < 0.001). The analysis results indicate that when users think that using PX Pay is convenient, they are likely to be more satisfied with related services and have a stronger intention to use PX Pay. H1 is thus supported (as shown in Figure 2). The expectation of ease of use had a significant positive effect on consumers’ behavioral intention to use PX Pay (β = 0.24, *t*-value = 3.794, *p*-value < 0.05). The results show that when users think that using PX Pay is easier, they have a stronger intention to use it. H2 is therefore supported.

Social influence had a significant positive effect on consumers’ behavioral intention to use PX Pay (β = 0.11, *t*-value = 1.94, *p*-value < 0.05). Thus, when users perceive the use of PX Pay to be affected by the environment, they have a stronger intention to use PX Pay. Thus, H3 is supported.

Behavioral intention had a significant positive effect on consumers’ behavior when using PX Pay (β = 0.25, *t*-value = 2.50, *p*-value < 0.01). The more willing users are to use PX Pay for payment and consumption activities, the higher the degree of usage behavior. H4a is therefore supported.

Facilitating conditions had no significant positive effect on consumers’ behavior in using PX Pay (β = 0.112, *t*-value = 1.217, *p*-value > 0.05). Thus, the personal resources, knowledge, or degree of assistance given to users did not affect their use of PX Pay. Hence, H4b was not supported.

Different levels of involvement had a significant positive effect on consumers’ behavioral intention to use PX Pay (β = 0.17, *t*-value = 2.10, *p*-value < 0.05). That is, higher degree of user involvement in PX Pay is associated with stronger intentions to use it. As a result, H5 is supported.

#### Hierarchical Regression Analysis

##### Analysis of the Effect of Gender Difference

**FIGURE 3:**
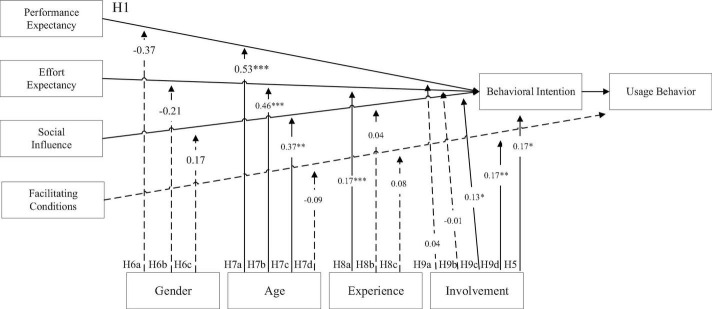
Moderation effects of UTAUT. *p < 0.05; **p < 0.01; ***p < 0.001.

First, the results of analyzing the various variables were tested with the Durbin-Watson statistic of the regression, which showed that the error terms were all independent of each other. Overall, the hierarchical regression model of behavioral intention, regardless of whether the independent variables were performance expectations (*R*^2^ = 0.48, *F* = 77.58, *p*-value < 0.001), ease-of-use expectations (*R*^2^ = 0.42, *F* = 61.10, *p*-value < 0.001) or social impact (*R*^2^ = 0.15, *F* = 14.96, *p*-value < 0.001), showed significant results. Furthermore, regression analysis was performed through a three-stage model to confirm H6a: performance expectations were used in model one (β = 0.67, *t*-value = 14.63, *p*-value < 0.001) and model two (β = 0.13, *t*-value = 2.86, *p*-value < 0.01) and model three (β = −0.37, *t*-value = −1.69, *p*-value > 0.05). To examine H6b, ease-of-use expectations were used in model one (β = 0.636, *t*-value = 13.21, *p*-value < 0.001), model two (β = 0.10, *t*-value = 2.22, *p*-value < 0.05) and model three (β = −0.21, *t*-value = −1.02, *p*-value > 0.05). To confirm H6c, social influence was used in model one (β = 0.38, *t*-value = 6.49, *p*-value < 0.001), model two (β = 0.08, *t*-value = 1.42, *p*-value > 0.05) and model three (β = 0.17, *t*-value = 0.79, *p*-value > 0.05). The interaction between gender and the independent variable had no significant effect, and there was no moderation effect, so the results of this study did not support H6a, H6b andH6c (as shown in Figure 3).

In addition, through independent-sample t-test analysis, it was further evaluated whether each aspect differed significantly in response intensity due to gender. The significance (*p*-value) of the effect of different genders for each aspect was greater than 0.05; thus, the impact was not significant. This type of personal characteristic has no effect on the behavioral intention-related factors related to using PX Pay. Thus, the results are insufficient to support H6a-H6c.

##### Analysis of the Effect of Age Difference

First, the Durbin-Watson statistic of regression (D-W = 2.23, 2.25, 2.28, 2.03) results showed that the error terms were all independent of each other. Overall, whether it is a hierarchical regression model of behavioral intention or usage behavior, when the independent variables are performance expectations (*R*^2^ = 0.50, *F* = 86.04, *p*-value < 0.001), ease-of-use expectations (*R*^2^ = 0.46, *F* = 71.38, *p*-value < 0.001), social influence (*R*^2^ = 0.200, *F* = 20.97, *p*-value < 0.001) or facilitating conditions (*R*^2^ = 0.07, *F* = 6.74, *p*-value < 0.001), the results are significant. Furthermore, regression analysis was performed through a three-stage model in order to confirm H7a: performance expectations were used in model one (β = 0.67, *t*-value = 14.63, *p*-value < 0.001), model two (β = −0.08, *t*-value = − 1.82, *p*-value > 0.05) and model three (β = 0.53, t-value = 4.62, *p*-value < 0.001). To confirm H7b, ease-of-use expectation was used in model one (β = 0.64, *t*-value = 13.21, *p*-value < 0.001), model two (β = −0.13, *t*-value = −2.77, *p*-value < 0.05) and model three (β = 0.46, *t*-value = 4.03, *p*-value < 0.001). To examine H7c, social influence was used in model one (β = 0.37, *t*-value = 6.49, *p*-value < 0.001), model two (β = −0.19, *t*-value = −3.302, *p*-value < 0.001) and model three (β = 0.37, *t*-value = 2.64, *p*-value < 0.01). To confirm H7d, facilitating conditions were used in in model one (β = 0.21, *t*-value = 3.48, *p*-value < 0.001), model two (β = 0.17, *t*-value = 2.75, *p*-value < 0.01) and model three (β = −0.086, *t*-value = −0.56, *p*-value > 0.05). The results show that the interaction between age and performance expectations, ease-of-use expectations, and social influence has a significant moderating effect, whereas the interaction with facilitating conditions had no significant moderating effect. Thus, H7a, H7b and H7c were all supported, but H7d was not supported.

To fully understand the influence of behavioral intentions and usage behavior related to PX Pay and whether response intensity would differ by age, this research used ANOVA to further explore their relevance. The analysis included performance expectations [*F*_(2_, _256)_ = 0.66, *p*-value = 0.52, ηp2 = 0.01], ease-of-use expectations [*F*_(2_, _256)_ = 0.03, *p*-value = 0.97, ηp2 = 0.00], social influence [*F*_(2_, _256)_ = 3.63, *p*-value = 0.03, ηp2 = 0.03] and facilitating conditions [*F*_(2_, _256)_ = 1.60, *p*-value = 0.20, ηp2 = 0.01]. The age factor had a significant impact only on social impact. The *p*-values for the other associations were all greater than.05, indicating that there was no significant impact.

Given that social influences had different but significant effects on behavioral intention on the basis of age difference (*F* = 3.63, *p*-value < 0.05), the Scheffé method was used as a postmortem empirical test, and the results showed that there is a significant difference between individuals over 50 and those aged 30–39 (*p*-value < 0.05). It can be inferred that the behavioral intention of using PX Pay is more likely to fluctuate for those over 50 years old due to their thoughts and perceptions of important relationships, followed by the 30–39 age group.

##### Analysis of the Moderation Effect of Use Experience

The Durbin-Watson statistic (D-W = 2.26, 2.28, 2.15) showed that the error terms were independent of each other. For a hierarchical regression model with either behavioral intention or usage behavior, the independent variables with significant effects were ease-of-use expectations (*R*^2^ = 0.46, *F* = 71.74, *p*-value < 0.001) and social influence (*R*^2^ = 0.26, *F* = 29.68, *p*-value < 0.001) or facilitating conditions (*R*^2^ = 0.07, I = 6.61, *p*-value < 0.001). Furthermore, regression analysis was performed through a three-stage model to examine H8a, with ease-of-use expectations in model one (β = 0.64, *t*-value = 13.21, *p*-value < 0.001), model two (β = 0.18, *t*-value = 3.45, *p*-value < 0.001) and model three (β = 0.17, *t*-value = 3.54, *p*-value < 0.001). To confirm H8b, social influence was in model one (β = 0.37, *t*-value = 6.49, *p*-value < 0.001), model two (β = 0.35, *t*-value = 6.34, *p*-value < 0.001) and model three (β = 0.04, *t*-value = 0.67, *p*-value > 0.05). To confirm H8c, facilitating conditions were used in model one (β = 0.21, *t*-value = 3.48, *p*-value < 0.001), model two (β = 0.16, *t*-value = 2.44, *p*-value < 0.05) and model three (β = 0.08, *t* < 0.24, *p*-value > 0.05). This result indicates that the interaction between use experience and ease-of-use expectations had a significant impact, and there was a moderation effect. However, the interaction with social influences and facilitating conditions had no significant effect, and there was no moderation. Therefore, H8a was supported in this study, but H8b and H8c were not.

To further explore use experience and how to produce different moderation effects between ease-of-use expectations and behavioral intention of using PX Pay, we followed the suggestions of Aiken et al. (1991) and substituted variables into the regression equation with a standard deviation value above and below the average and an interaction effect. When the user experience status was high, ease-of-use expectations had a stronger influence on behavioral intention. However, the degree of influence was relatively small, and it was confirmed that the moderation effect was indeed significantly in terms of the influence and predictive power among the variables.

##### Analysis of the Moderation Effect of Degree of Involvement

The Durbin-Watson statistic test (D-W = 2.24, 2.23, 2.30, 2.12) shows that the error terms were independent of each other. Whether a hierarchical regression model uses behavioral intention or usage behavior, when the independent variables were performance expectations (*R*^2^ = 0.49, *F* = 80.38, *p*-value < 0.001), ease-of-use expectations (*R*^2^ = 0.43, *F* = 63.26, *p*-value < 0.001), social influence (*R*^2^ = 0.19, *F* = 19.49, *p*-value < 0.001) or facilitating conditions (*R*^2^ = 0.08, *F* = 7.68, *p*-value < 0.001), all results were significant. Furthermore, regression analysis was performed through a three-stage model to confirm H9a, with performance expectations in model one (β = 0.67, *t*-value = 14.63, *p*-value < 0.001) and model two (β = 0.18, *t*-value = 3.87, *p*-value < 0.001) and model three (β = 0.04, *t*-value = 0.84, *p*-value > 0.05). To confirm H9b, ease-of-use expectations were used in model one (β = 0.64, *t*-value = 13.21, *p*-value < 0.001), model two (β = 0.16, *t*-value = 3.14, *p*-value < 0.01) and model three (β = −0.01, *t*-value = −0.090, *p*-value > 0.05). To confirm H9c, social influence was used in model one (β = 0.38, *t*-value = 6.49, *p*-value < 0.001), model two (β = 0.22, *t*-value = 3.08, *p*-value < 0.01) and model three (β = 0.13, *t*-value = 2.12, *p*-value < 0.05). Finally, to confirm H9d, facilitating conditions were used in model one (β = 0.21, *t*-value = 3.48, *p*-value < 0.001), model two (β = 0.12, *t*-value = 1.79, *p*-value > 0.05) and model three (β = 0.17, *t*-value = 2.69, *p*-value < 0.01). The results show that the interactions between degree of involvement and performance expectations and ease-of-use expectations had no significant impact or moderation effect. However, the interactions with social influences and facilitating conditions did have a significant impact and moderation effect. Hence, H9a and H9b were not supported, but both H9c and H9d were supported.

To gain a deeper understanding of the degree of involvement and what influences the main relationship, the research approach of Aiken et al. (1991) was used, and an interaction effect diagram was created. It was concluded that when the degree of involvement was high, social influence had a stronger influence on behavioral intention; otherwise, the degree of influence was small. On the other hand, when the degree of involvement was low, facilitating conditions had a greater impact on behavior; thus, the impact was stronger.

## Conclusion and Suggestions

### Summary and Discussion of Research Results

This study proposes a total of 20 hypotheses, of which 11 hypotheses were supported and 9 were not supported. The results of the analysis will be explained in the following order. The path diagram of the relationships in this research is shown below:

First, the empirical results show that when users believe that using PX Pay can achieve their goal of convenience or increase their satisfaction with related services, their behavioral intention to use PX Pay is increased. The results are consistent with the original theoretical assumptions of UTAUT (Venkatesh et al., 2003), and past studies have also noted the importance of performance expectations in the acceptance of technology (Davis, 1989; Venkatesh and Davis, 2000; Oechslein et al., 2014), which also confirms that the relationship between performance expectations and behavioral intentions is consistent. When users think that PX Pay is easier to use, they have stronger intentions to adopt it. According to Park and Keil (2019) research, if consumers think that mobile marketing tools are simple and easy to use, they can also find the information they need without spending extra time and energy, which makes mobile marketing more acceptable. Behavioral intention may increase accordingly. The clear and easy-to-understand interface and function design of PX Pay allows users to quickly get started and familiarize themselves with its operations, thus strengthening their behavioral intention.

Furthermore, consumers who are more affected by their surroundings have a stronger intention to adopt PX Pay, and this result is consistent with the results of past research. If consumers perceive that using mobile marketing tools will help others around them (friends, relatives, media) evaluate and perceive them more positively, their degree of behavioral intentions will further increase (Park and Keil, 2019). Recall that PX Mart adopted a member recommendation strategy in the early stage of PX Pay implementation. Through the power of social influence, the app quickly achieved outstanding results in cumulative app downloads. behavioral intention has a positive effect on usage behavior. That is, the stronger the user’s intention to use PX Pay is, the higher their actual frequency of use. The UTAUT-related literature has also pointed out that behavioral intentions have a significant impact on actual technology adoption behavior (Venkatesh et al., 2003). Therefore, when users have a high willingness to use PX Pay, they usually actually do use the system. Therefore, PX Mart can consider how to use incentive factors/mechanisms to improve behavioral intention and thus drive usage behaviors and increase usage frequency.

However, facilitating conditions had no significant impact on the use of PX Pay. This result is completely different from the original hypothesis of UTAUT and also differs from ordinary people’s perception. This article infers that because Taiwan’s mobile payment options are diverse, PX Pay does not yet have a high degree of user loyalty, and users can still choose other payment options. Therefore, even if a user has a full understanding of how to use PX Pay, this may not necessarily improve the actual frequency of use. Finally, the degree of involvement positively affects consumers’ behavioral intentions regarding PX Pay, and this is consistent with the research results of Cai et al. (2017). That is, when consumers are willing to spend more time understanding PX Pay, they have a higher degree of involvement and thus a stronger willingness to use the product/service.

The following descriptions is about the moderation effects of gender, age, use experience and degree of involvement. First, according to the research results, gender had no effect on the behavioral intention to use PX Pay among performance expectations, ease-of-use expectations and social influence. A possible reason is that the products sold by the PX Mart Channel have no obvious gender orientation. In addition, today’s app development and design tend to be easy to use, and ease of use is the highest objective. Previous literature also indicates that women are more susceptible to social influences when adopting new technologies. It is speculated that since PX Mart’s promotional activities in the initial stage of PX Pay introduction were very effective, this not only generated remarkable rates of downloads but also significantly improved the product image of PX Pay from various gender perspectives. In terms of the degree of satisfaction, practical assistance, difficulty in operation, and the influence of important others, response intensity does not differ significantly between men and women.

Age had a moderating effect on the behavioral intention to use PX Pay among performance expectations, ease-of-use expectations and social influence. This conclusion is consistent with the integrated technology acceptance model (Venkatesh et al., 2003). Echoing the arguments and the empirical results of this research, the findings show that the social influence on PX Pay customers over 50 years old is greater than that on PX Pay customers over 30–39 years old. Elderly individuals are more likely to be affected by the perception of important relationships. Influencing usage intention is consistent with Park and Keil (2019) research results suggesting that consumers’ intention to operate consumer electronic products—such as tablet computers—is most affected by relatives, friends, and media advertisements. In addition, according to previous literature, elderly people tend to value facilitating conditions when considering whether to adopt new technologies. Therefore, this article speculates that since PX Mart directly adopted the promotion model of on-site teaching via store personnel in the initial stage of PX Pay introduction, users of different ages have been exposed to similar facilitating conditions. As a result, age did not moderate the relationship between facilitating conditions and usage behavior.

Furthermore, use experience moderated the relationship between ease-of-use expectations and behavioral intention to use PX Pay. The results are in line with expectations and echo the empirical evidence provided by past experts and scholars. When using a new system, users consider their own experience. Experience determines behavioral intentions and usage behaviors, and the ease-of-use expectations of information systems also change with the accumulation of user experience (Venkatesh and Davis, 2000; Venkatesh et al., 2003). According to Shen et al. (2011), the effect of social influence will no longer be significant as use experience increases. This research suggests that this is an effect of PX Mart’s strong promotional activities in the initial stage of PX Pay introduction; even if the social impact is extremely high, there has been no major negative review thus far. There is no significant difference in use experience due to the length of time; according to the study of Bergeron et al. (1990), as use experience increases, facilitating conditions increase. Therefore, the reasoning in this research is still that PX Mart’s direct promotion of on-site teaching by store personnel in the early stage of PX Pay introduction enabled consumers to quickly obtain the ability to operate the app, making user experience less of an issue. There are obvious differences over time, so there is no moderation effect between facilitating conditions and usage behavior.

Finally, the degree of involvement has a moderating effect between social influence and behavioral intention to use PX Pay, and there is a moderating effect on the relationship between facilitating conditions and usage behavior. The research hypothesis is supported by empirical evidence and the results of Xiao (2008). Correspondingly, the higher the user’s involvement is, the greater the effect of social influence on behavioral intention. However, when the user’s involvement is low, facilitating conditions have a stronger influence on behavior. We speculate that because the main use scenario of PX Pay is payment in physical channels and PX Mart does not force shoppers to use PX Pay, consumers can still use other payment methods that are relatively familiar and comfortable for them. The degree of involvement had no moderating effect on the relationship between performance expectations and ease-of-use expectations for behavioral intention for using PX Pay.

### Theoretical Contributions and Implications

The theoretical contribution of this research is to confirm the three dimensions of UTAUT, namely, performance expectations, social impact and ease-of-use expectations. This knowledge can be used to improve the motivation of middle-age and older consumers, in turn affecting their usage behavior, but facilitating conditions were found to be more important for middle-age and older consumers. Motivation for use had no significant effect. In addition, it was confirmed that the degree of involvement affected the motivation of middle-age and older consumers. Therefore, this study advocates that when using the UTAUT model, the influence of marketing behaviors on technology users should be considered, and the degree of involvement factor should be added to the model. Future researchers may perform more in-depth analysis of data from middle-age and older consumers.

### Research Limitations and Future Works

This study was limited by manpower, time, and funding, and had the following research limitations. First, this study used an online questionnaire to conduct the sample survey. In addition to the fact that it is difficult to attract users to fill out the questionnaire through online interaction, there is still room for discussion on the truthfulness and purpose of the questionnaire. In the future, we can collect more samples if we can conduct activities or cooperate more deeply with related industries. However, this study has actively screened the returned questionnaires, and the characteristics of the sample are generally consistent with the characteristics of PX Mart’s main mobile payment customers, so the study’s findings still have practical reference value. Second, consumers’ acceptance of non-cash transactions has gradually increased in recent years, and this study shows that middle-aged and elderly consumers are also beginning to accept mobile payments. Future studies may focus on the digital transformation strategies of supermarkets, from physical e-commerce models to online involvement and continuous involvement. Because of Covid-19, more and more people have recently started to purchase food items such as fruit and vegetable boxes or frozen seafood and meat online, which will increase the chances of consumers adopting mobile payment. However, researchers still need to study whether this change in consumption patterns and Internet habits is just a short-term phenomenon or a sustainable long-term trend. Therefore, it is also worth exploring how to match different sales strategies to increase customers’ usage and stickiness on the shopping platform in the future.

## Data Availability Statement

The raw data supporting the conclusions of this article will be made available by the authors, without undue reservation.

## Author Contributions

C-HL, Y-TC, and TH: conceptualization. C-HL, Y-TC, and SK: methodology. Y-JC: investigation. Y-TC and Y-JC: formal analysis. SK and TH: supervision and visualization. Y-TC: validation. C-HL, Y-TC, SK, and TH: writing—original draft preparation and writing—review and editing. All authors have read and agreed to the published version of the manuscript.

## Conflict of Interest

The authors declare that the research was conducted in the absence of any commercial or financial relationships that could be construed as a potential conflict of interest.

## Publisher’s Note

All claims expressed in this article are solely those of the authors and do not necessarily represent those of their affiliated organizations, or those of the publisher, the editors and the reviewers. Any product that may be evaluated in this article, or claim that may be made by its manufacturer, is not guaranteed or endorsed by the publisher.
